# SUMO-4: A novel functional candidate in the human placental protein SUMOylation machinery

**DOI:** 10.1371/journal.pone.0178056

**Published:** 2017-05-17

**Authors:** Dora Baczyk, Melanie C. Audette, Sascha Drewlo, Khrystyna Levytska, John C. Kingdom

**Affiliations:** 1 Program in Development and Fetal Health, Lunenfeld–Tanenbaum Research Institute, Mount Sinai Hospital, Toronto, Canada; 2 Faculty of Medicine, University of Toronto, Toronto, Canada; 3 Department of Obstetrics and Gynecology, Wayne State University School of Medicine, Detroit, Michigan, United States of America; 4 Department of Laboratory Medicine and Pathobiology, University of Toronto, Toronto, Canada; 5 Maternal-Fetal Medicine Division, Department of Obstetrics and Gynecology, Mount Sinai Hospital, Toronto, Canada; 6 Department of Obstetrics and Gynecology, University of Toronto, Toronto, Canada; German Cancer Research Center, GERMANY

## Abstract

**Background:**

Small ubiquitin-like modifiers (SUMOs) conjugate to proteins post-translationally, thereby affecting target localization, activity and stability. Functional SUMO family members identified in the human placenta include SUMO-1 to SUMO-3, which are elevated in pre-eclampsia. Whether the fourth isoform, SUMO-4, plays a role in placental development and function remains unknown.

**Objectives:**

We tested the hypothesis that SUMO-4 is expressed in the human placenta and demonstrates altered SUMOylation in pre-eclamptic pregnancies.

**Methods:**

SUMO-4 mRNA (qRT-PCR) and protein (Western blot and immunohistochemistry) were measured in Jar cells, BeWo cells, first trimester placental villous explants and placental tissues across normal gestation and in pre-eclampsia. SUMO-4 expression in response to oxidative stress (H_2_O_2_: 0, 0.1, 1 and 5mM), as well as, hypoxia-reperfusion (O_2_: 1%, 8% and 20%) was measured. Lastly, SUMO-4 binding (covalently vs. non-covalently) to target proteins was investigated.

**Results:**

SUMO-4 mRNA and protein were unchanged across gestation. SUMO-4 was present in the villous trophoblast layer throughout gestation. SUMO-4 mRNA expression and protein levels were increased ~2.2-fold and ~1.8-fold in pre-eclamptic placentas compared to age-matched controls, respectively (p<0.01). SUMO-4 mRNA and protein expression increased in Jars, BeWos and first trimester placental explants with 5mM H_2_O_2_ treatment, as well as with exposure to hypoxia-reperfusion. SUMO-1 to SUMO-3 did not show consistent trends across models. SUMO-4 hyper-SUMOylation was predominantly covalent in nature.

**Conclusions:**

SUMO-4 is expressed in normal placental development. SUMO-4 expression was increased in pre-eclamptic placentas and in models of oxidative stress and hypoxic injury. These data suggests that SUMO-4 hyper-SUMOylation may be a potential post-translational mechanism in the stressed pre-eclamptic placenta.

## Introduction

SUMOylation is a post-translational process in which small ubiquitin-like modifiers (SUMOs) are covalently conjugated to target proteins by the enzyme UBC9. SUMOylation acts in a number of ways to regulate cellular signaling including its affects on target protein function, localization and stability, as well as, DNA repair and cell cycle progression [1]. SUMO proteins can also be removed (deSUMOlyation) by the sentrin-specific proteases (SENPs). These enzymes use their isopeptidase activity to cleave the covalent bond between the SUMO and its target [2]. In addition to covalent modifications, SUMOs are able to post-translationally modify targets by forming a non-covalent interaction via a SUMO interacting binding motif (referred to as SIM/SBM) [3]. As a result, this non-covalent association gives rise to a novel binding site for a third interacting protein [4].

Four SUMO isoforms (SUMO-1, SUMO-2, SUMO-3 and SUMO-4), have thus far been identified in humans. SUMO proteins share homology between isoforms, with the greatest being between that of SUMO-2 and SUMO-3 (97% homologous) [5]. With such a large homologous sequence, it is often difficult to distinguish between these two isoforms, and as such, they are commonly examined in conjunction as SUMO-2/3. The first three SUMOs are constitutively expressed in all eukaryotic cells, while by contrast SUMO-4 has a unique distribution. To date, SUMO-4 has only been detected in renal, immune and pancreatic cells [6–8].

SUMOylation is known to be a fundamental cellular process required for placental development and function. Knocking out SENP1 and SENP2 (deSUMOylating enzymes) in transgenic mouse models results in pregnancies with non-viable embryos and impaired cell cycle progression, proliferation and differentiation of placental trophoblasts [9,10]. Our group has previously demonstrated that SUMO-1, SUMO-2, SUMO-3 and UBC9 (SUMO conjugating enzyme) are found in the human placenta across gestation [11]. Furthermore, evidence suggests that not only are SUMOs required for normal placental function, they are also implicated in the obstetrical complication of pre-eclampsia (PE). Hyper-SUMOylation is reported in PE, with increased mRNA and protein expression of placental SUMO-1, SUMO-2/3 and UBC9 [11]. Furthermore, hypoxia has shown to upregulate SUMO-1, SUMO-2, SUMO-3 and UBC9 in first trimester explants [11], supporting the role of SUMOylation in severe PE, which is often characterized by placental ischemic reperfusion injury [12].

SUMO isoforms 1 to 3 and UBC9 were previously suggested to participate in the pathogenesis of placental dysfunction underlying PE, though the potential role of SUMO-4 is presently unknown. In this study, we tested the hypothesis that SUMO-4 isoform is present in the human placenta and its expression is altered in PE. As PE placentas are typically exposed to excessive oxidative stress via ischemic injury [12], the effects of H_2_O_2_ treatment and hypoxia-reperfusion on SUMO-4 in placental models were also investigated.

## Methods

### Tissue collection

First and second trimester placental tissues were obtained following voluntary pregnancy terminations (Morgentaler Clinic; Toronto, Canada). Preterm age-matched controls and PE placental tissue was obtained from the Placental BioBank (Mount Sinai Hospital; Toronto, Canada) following deliveries that occurred in singleton pregnancies between 24^+0^ and 34^+6^ weeks gestation. Selection criteria for PE was based of the American Congress of Obstetricians and Gynecologists (ACOG) guidelines, which defined PE as new onset (>20 weeks gestation) either: 1. hypertension (>140/90 mmHg) with proteinuria (>1+ on dipstick, protein/creatinine ratio >0.3 or >300 mg/24 h) or associated features (thrombocytopenia, renal insufficiency, impaired liver function, pulmonary edema or cerebral or visual symptom) and delivered <34^+0^ weeks gestation OR 2. hypertension (>160/110 mmHg) with proteinuria or associated features (previously listed) and delivered <34^+6^ weeks gestation [13]. Pre-term controls were selected based on a birthweight >20^th^ centile, normal umbilical artery Doppler, normal blood pressure (<140/90) and no gestational diabetes or chorioamnionitis [14]. Maternal demographic information for preterm age-matched controls and PE tissue is provided in Czikk *et al*. (2013). All patients gave written informed consent and the Research Ethics Board approval was obtained from Mount Sinai Hospital (MSH, REB#11-0248-E). All placental samples were studied in biological triplicates.

### Oxidative stress by H_2_O_2_ and hypoxia treatment in Jars, BeWos and placental explants

In addition to placental explants, placental Jar and BeWo cells were used to investigate oxidative stress on levels of SUMO-4. BeWo cells were cultured as previously described [15] and Jar cells were cultured in RPMI-1640 media (Gibco—Life technologies corporation, Grand Island NY, USA) supplemented with 5% FBS (Wisent, Canada), penicillin/streptomycin (Life Technologies), 4500mg glucose/L (Sigma, Oakville, ON, Canada) and 10mM Hepes (Sigma). First trimester placental explants (7–8 weeks) were cultured as described previously [16]. H_2_O_2_ treatment of placental cells and explants was done at 0 (control), 0.1, 1 and 5mM for a 24 hours (h) incubation period. To investigate the effects of ischemic-reperfusion injury, explants were cultured overnight in a normoxic (8% O_2_) environment and subsequently placed in either hypoxic (1% O_2_), normoxic (8% O_2_), hyperoxic conditions (20% O_2_) or alternating conditions (cycled 1% and 20% O_2_ for 1h intervals), each for a total period of 8h. Following treatment, cells and explants were collected for RNA or protein extraction.

### SUMO mRNA expression by qRT-PCR

Total RNA was extracted from placental Jar and BeWo cells using RNeasy Plus Mini Kit (Qiagen, Mississauga, ON, Canada). Total RNA was extracted from placental tissues using RNeasy Plus Universal Kit (Qiagen). RNA was treated with gDNA elimination solution (Qiagen) prior to reverse-transcription using iScript^™^ Reverse Transcription Supermix (Bio-Rad, Mississauga, ON, Canada) according to manufacturer’s instructions. Real-time qPCR was conducted in triplicates using LuminoCt SYBR Green qPCR Ready Mix (3μl; Sigma-Aldrich) and primers (30nM) and cDNA (10ng) using the CFX384 Real-Time PCR Detection System (Bio-Rad). Primer sequences unless otherwise indicated are listed in [11]. Primer efficiencies ranged from to 95–111% and all CT values were between 20–30 cycles. The expression of genes of interest was normalized to the housekeeping genes TBP (Forward: TGC-ACA-GGA-GCC-AAG-AGT-GAA, Reverse: CAC-ATC-ACA-GCT-CCC-CAC-CA), YWHAZ and CYC1 for placental tissue and TBP, YWHAZ and HPRT (Forward: TGA-CAC-TGG-CAA-AAC-AAT-GCA, Reverse: GGT-CCT-TTT-CAC-CAG-CAA-GCT) for Jar and BeWo cells. Housekeeping genes were based on [17]. mRNA values were expressed as fold change relative to controls (set as value 1).

### SUMO protein expression by Western blotting

Following treatment, placental tissues were collected and snap-frozen using liquid nitrogen. In preparation for protein extraction, placental tissues were placed in boiling lysis high SDS buffer (10% glycerol, 1% SDS, 80mM Tris pH 6.8, 10mM NEM; Sigma) and phosphatase and protease inhibitors. Samples were homogenized using the Bullet Blender Blue Homogenizer (Ideal Scientific, Ancaster, ON, Canada). 25 μg of protein was boiled with 10% ß-mercapthoethanol for 10 min and electrophoresed in 4–20% Mini-PROTEIN TGX^™^ pre-cast gels (Bio-Rad). Proteins were then transferred to PVDF membrane (0.2μm) using Trans-Blot Turbo^™^ transfer pack (Bio-Rad) according to manufacturer’s instructions. Membranes were blocked (5% milk/TBST) and then incubated in primary antibodies overnight at 4°C (SUMO-1, 1:1000 (Abcam, MA, USA); SUMO-2/3, 1:500 (Abcam); SUMO-4, 1:3000 (Abcam); Lamin B, 1:500 (Santa Cruz); α-Tubulin, 1:5000 (Santa Cruz), and β-actin, 1:5000 (Santa Cruz)). Membranes were washed and incubated in appropriate secondary HRP-conjugated antibodies (GE Healthcare UK limited, UK). Membranes were developed using Western Lightning Plus-ECL (Thermo Scientific, Ottawa, ON, Canada) on autoradiography film (Denville Scientific, South Plainfield, NJ, USA). Quantity One Software (Bio-Rad) was used to quantify band intensities (within linear range). Proteins of interest were normalized to housekeeping proteins (α-Tubulin or β-actin). Protein values were compared to respective controls (set as 1).

### SUMO localization by immunohistochemistry

Placental tissues from healthy first trimester, second trimester and term, as well as, pre-eclamptic and pre-term age-matched controls were fixed in paraformaldehyde (4%) and wax-embedded. Sections were rehydrated and immunohistochemistry was performed as described previously in [11]. Sections were incubated with SUMO-4 primary antibodies at 1:500 dilution (Abcam) overnight at 4°C. The following day, the secondary biotinylated antibody (1:300; Dako, Carpinteria, CA, USA) was incubated for 1h at room temperature followed by treatment with streptavidin-HRP (1:2000, Invitrogen, Burlington, ON, Canada) for 1h at room temperature. Negative controls omitted the use of primary antibody and the incubation of non-specific IgG (data not shown). Slides were counter-stained with hematoxylin (Sigma). A Nikon DMRX light microscope was used and images were taken using a Sony PowerHAD 3CCD color video camera DXC-970ND (Sony, Toronto, ON, Canada). Four biological replicates were performed in each grouping.

### SUMO-4 interactions in BeWo cells

BeWo cells were stressed by FBS deprivation for 24h to induce global SUMOylation. Protein was isolated using high SDS buffer (as described above). To examine covalent interactions of SUMO-4, high SDS buffer was boiled with the addition of 10% ß-mercaptoethanol for 10 min at 100°C. This treatment was then compared to the preparation of proteins in ice-cold, low SDS-RIPA buffer (25mM Tris pH 7, 150mM NaCl, 0.1% SDS, 0.5% sodium deoxycholate, 0.1% Triton X-100, 10mM NEM). The use of high SDS-RIPA buffer results in denaturation of isopeptidases and thus elimination of non-covalent interactions, enriching the detection of covalent SUMO interactions. SUMO-4 levels in covalent and non-covalent preparations were compared using Western blotting (as described above).

### Statistical analysis

SUMO-4 mRNA and protein expression across gestation were analyzed using one-way ANOVA with Bonferroni’s multiple comparisons post-hoc test. Student’s t-test was used to compare SUMO-4 mRNA and protein levels in age-matched preterm controls vs. PE placental tissues. A two-way ANOVA with Bonferroni post-hoc analysis comparing all columns to relative vehicle control (1.0) was used to measure mRNA expression in Jars, BeWos, and first trimester placental explants. All statistical analyses were performed using GraphPad Prism 4 software (Graph Pad Prism, Inc, CA, USA). Significance was considered at P≤0.05. Data are represented as mean + standard error of the mean.

## Results

### SUMO-4 expression across gestation and in pre-eclampsia

**Fig 1:**
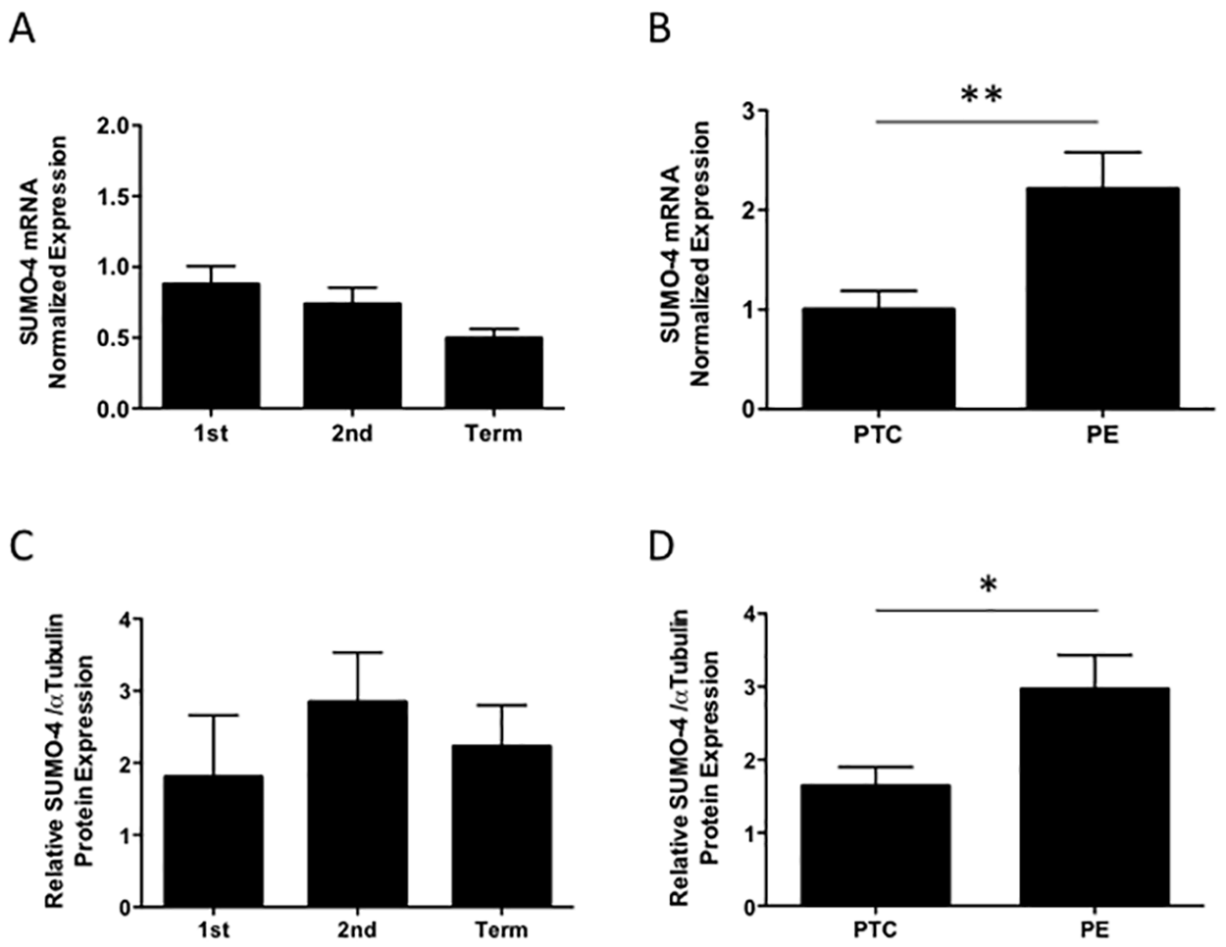
SUMO-4 mRNA and protein levels across gestation and in pre-eclampsia (PE). SUMO-4 (A) mRNA (n = 8–15) and (C) protein expression was unchanged across gestation (n = 3–6). Conversely, PE placentas showed elevated SUMO-4 (B) mRNA (**p<0.01, n = 4–6) and (D) protein (*p<0.05, n = 11–13) relative to pre-term age matched controls (PTC; set as 1). 1^st^ = first trimester, 2^nd^ = second trimester. Values represented as mean+SEM.

**Fig 2:**
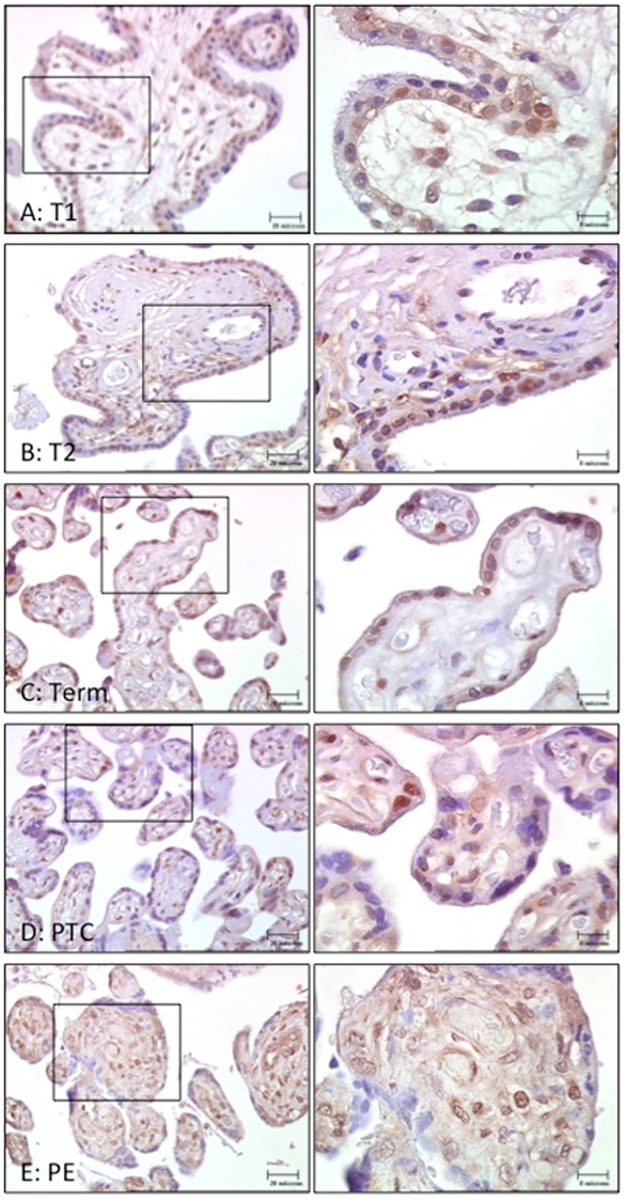
SUMO-4 immuno-staining in placentas from: (A) First-trimester (T1), (B) Second trimester (T2), (C) Term, (D) Preterm age-matched controls (PTC) and (E) Pre-eclampsia (PE). SUMO-4 was present in the villous trophoblast layer across gestation. PE placentas showed strong immuno-staining throughout the trophoblast and stroma compared to PTC. Images in left panels are presented at 40x magnification and boxed areas demonstrate images in right panels presented at 100x magnification.

*SUMO-4* mRNA was expressed constitutively across gestation (Fig 1A). More notably, *SUMO-4* mRNA levels were significantly increased in PE placentas compared preterm age-matched controls (2.2-fold, p<0.01, n = 6; Fig 1B). SUMO-4 protein levels also remained unchanged across gestation (Fig 1C), and similarly elevated in PE placentas compared to preterm age-matched controls (1.8-fold, p<0.05, n = 11–13; Fig 1D). Immuno-staining for SUMO-4 revealed expression predominantly in the trophoblast layer across gestation (Fig 2A–2C). Staining in PE placentas demonstrated strong expression throughout both the trophoblast and the stroma compared to preterm age-matched controls (Fig 2D and 2E).

### H_2_O_2_ induced oxidative stress in placental cell lines and first trimester placental explants

**Fig 3:**
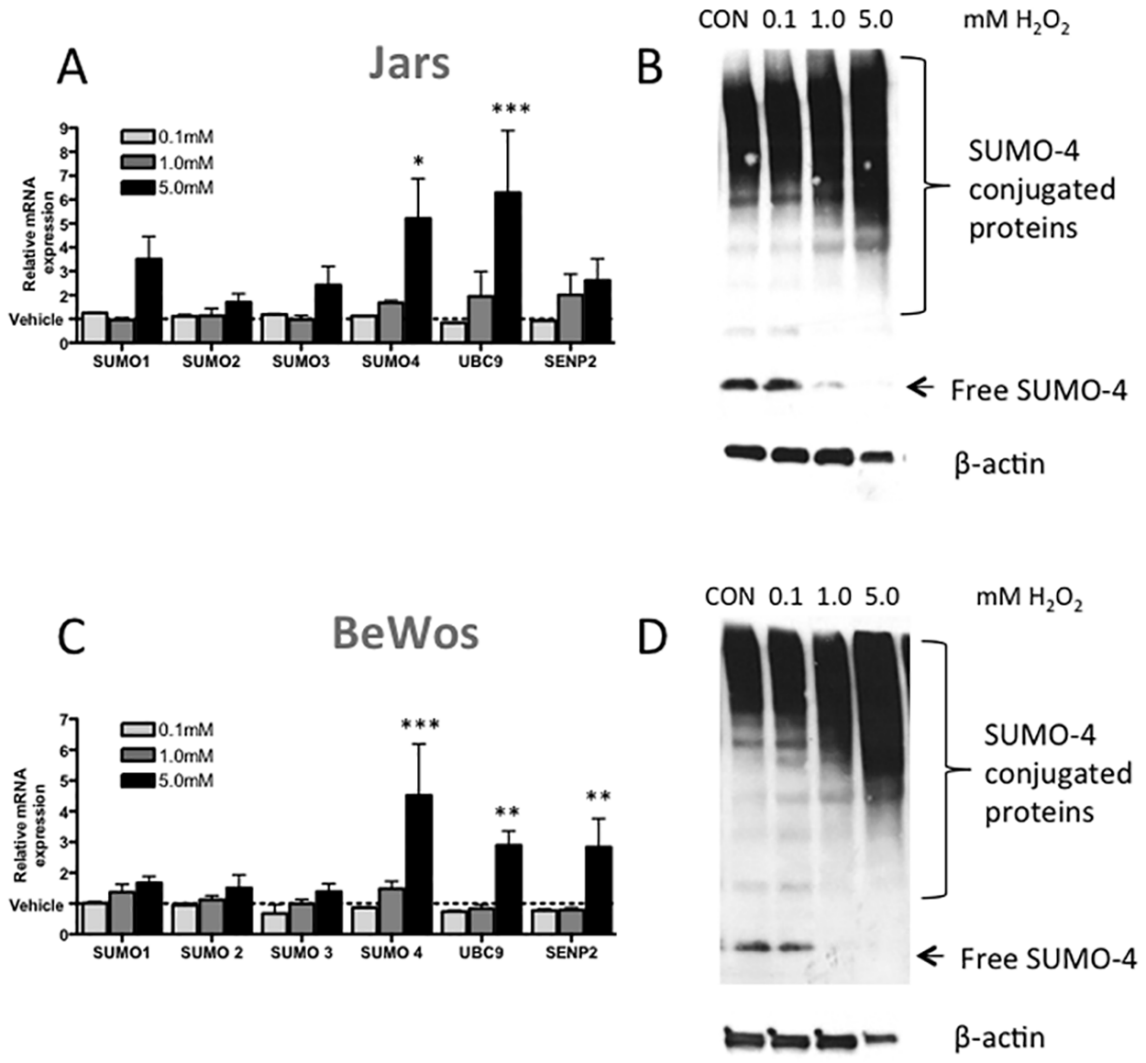
H_2_O_2_ treatment of placental (A & B) Jar and (C & D) BeWo cells induces SUMOylation at the (A & C) mRNA and (B & D) protein level (representative Western blots). Treatment of Jar cells with 5mM of H_2_O_2_ (24h) induced *SUMO-4* and *UBC9* mRNA, as well as SUMO-4 protein expression. Treatment of BeWo cells also induced *SUMO-4*, *UBC9* and *SENP2* mRNA levels and SUMO-4 protein expression. Values represented as mean+SEM, n = 3–4, Significance ***p<0.001, **p<0.01, *p<0.05. CON = control.

Treatment with 5mM of H_2_O_2_ for 24h strongly induced *SUMO-4* mRNA expression by 5.2±1.7 fold in Jar cells (p<0.05, n = 5; Fig 3A) and in BeWo cells by 4.5±2.9 fold (p<0.001, n = 4; Fig 3C). *UBC9* was increased in Jar (6.29±2.6 fold, p<0.001; Fig 3A) and BeWo (2.90±0.5 fold, p<0.01; Fig 3C) cells after 5mM H_2_O_2_ treatment. *SENP2* mRNA expression was increased with 5mM H_2_O_2_ treatment in BeWo cells (2.84±0.9 fold, p<0.01; Fig 3C). Western blot analysis revealed a similar dose-response increase of SUMO-4 conjugation to target proteins with increasing H_2_O_2_ concentrations in Jar and BeWo cells. This increase in conjugation corresponded with a decrease in free SUMO-4 protein (Fig 3B and 3D).

**Fig 4:**
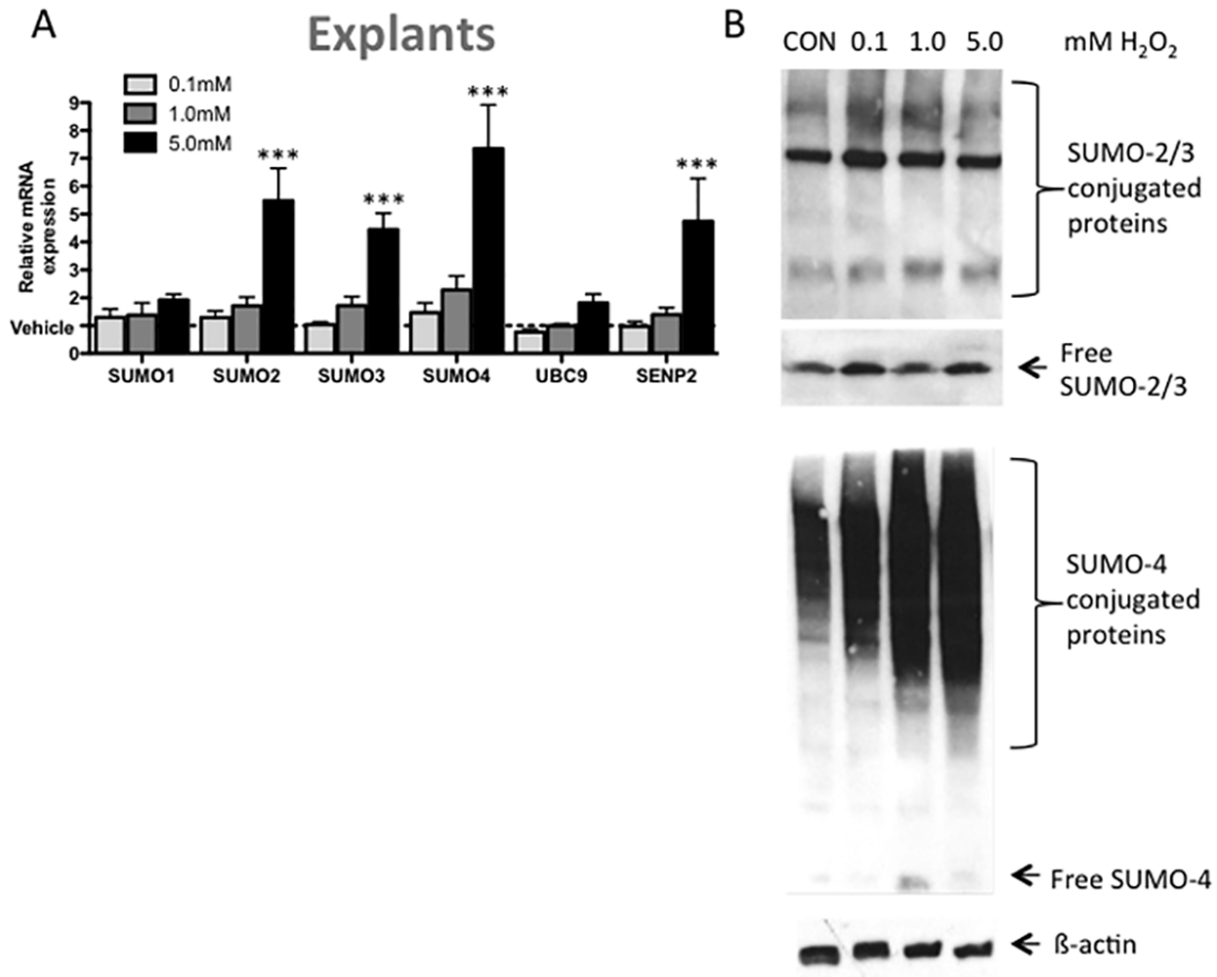
H_2_O_2_ treatment of first trimester placental explants induces SUMOylation at the (A) mRNA and (B) protein level. H_2_O_2_ treatment for 24h induced *SUMO-2*, *SUMO-3*, *SUMO-4* and *SENP* mRNA levels (compared to vehicle control). (B) Representative Western blots shown of SUMO-2/3 and SUMO-4. SUMO-2/3 protein remains unchanged with treatment, however SUMO-4 protein expression is increased. Values represented as mean+SEM; n = 5, Significance ***p<0.001. CON = control.

5mM H_2_O_2_ treatment for 24h upregulated *SUMO-2* (5.5±1.2 fold), *SUMO-3* (4.5±0.6 fold), *SUMO-4* (7.4±1.6 fold) and *SENP2* (4.75±1.5 fold) mRNA in first trimester placental explants (p<0.001, n = 5; Fig 4A). Conjugated and free protein levels of SUMO-2/3 remained unchanged with H_2_O_2_ treatment. However, SUMO-4 protein conjugation increased with the oxidative stress of H_2_O_2_ (Fig 4B).

### Hypoxia-reperfusion induced SUMO-4 levels in first trimester placental explants

**Fig 5:**
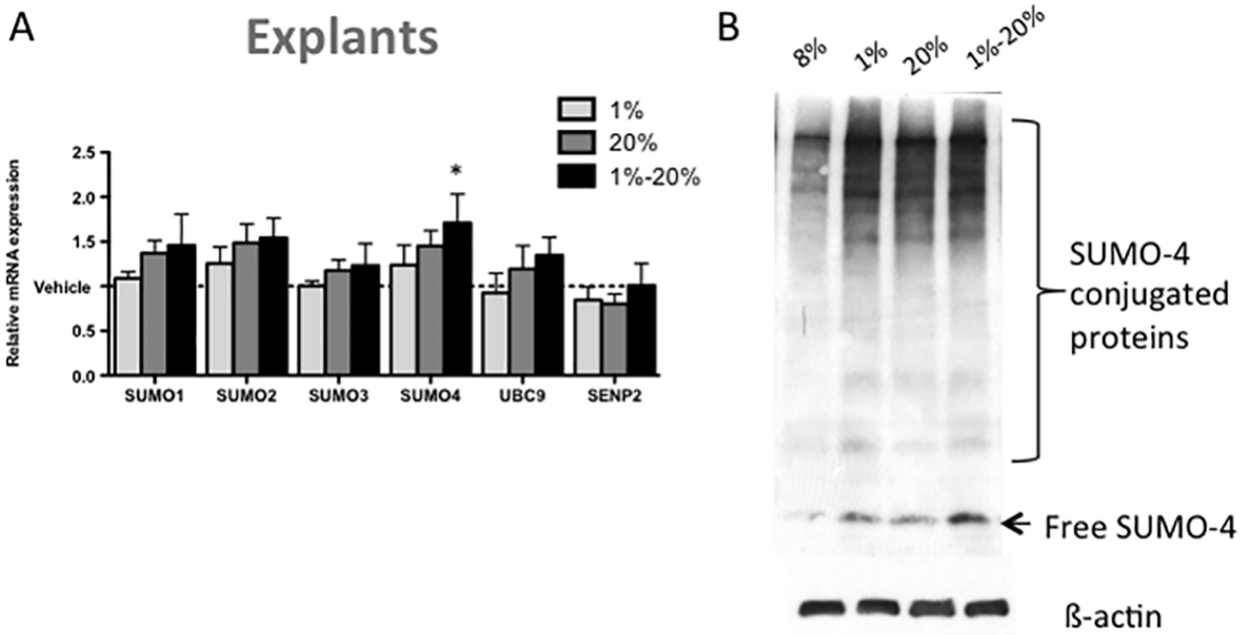
Hypoxia-reperfusion stress in first trimester placental explants induced SUMOylation of SUMO-4 at the (A) mRNA and (B) protein level. Explants were treated with hypoxia (1%), hyperoxia (20%) or hypoxia-reperfusion (cycled between 1% and 20% for 1h intervals) for a total of 8h. (A) Hypoxia-reperfusion induced *SUMO-4* mRNA expression relative to vehicle control (normoxic 8% O_2_). (B) Representative images of SUMO-4 Western blotting. SUMO-4 protein conjugation is increased with oxygen stress. Values represented as mean+SEM; n = 5, Significance *p<0.05.

Treatment of first trimester placental explants with hypoxia (1% O_2_) and hyperoxia (20% O_2_) for 8h did not affect *SUMO-1 to SUMO-4*, *UBC9 or SENP2* mRNA expression (Fig 5A). However, alternating cycles of hypoxia and hyperoxia for 8h increased *SUMO-4* mRNA expression by 1.71±0.32 fold (p<0.05; Fig 5A). SUMO-4 protein conjugation also increased with oxygenation stress in hypoxic, hyperoxic and hypoxia-reperfusion treatment (Fig 5B).

### SUMO-4 interacts covalently with target proteins

**Fig 6:**
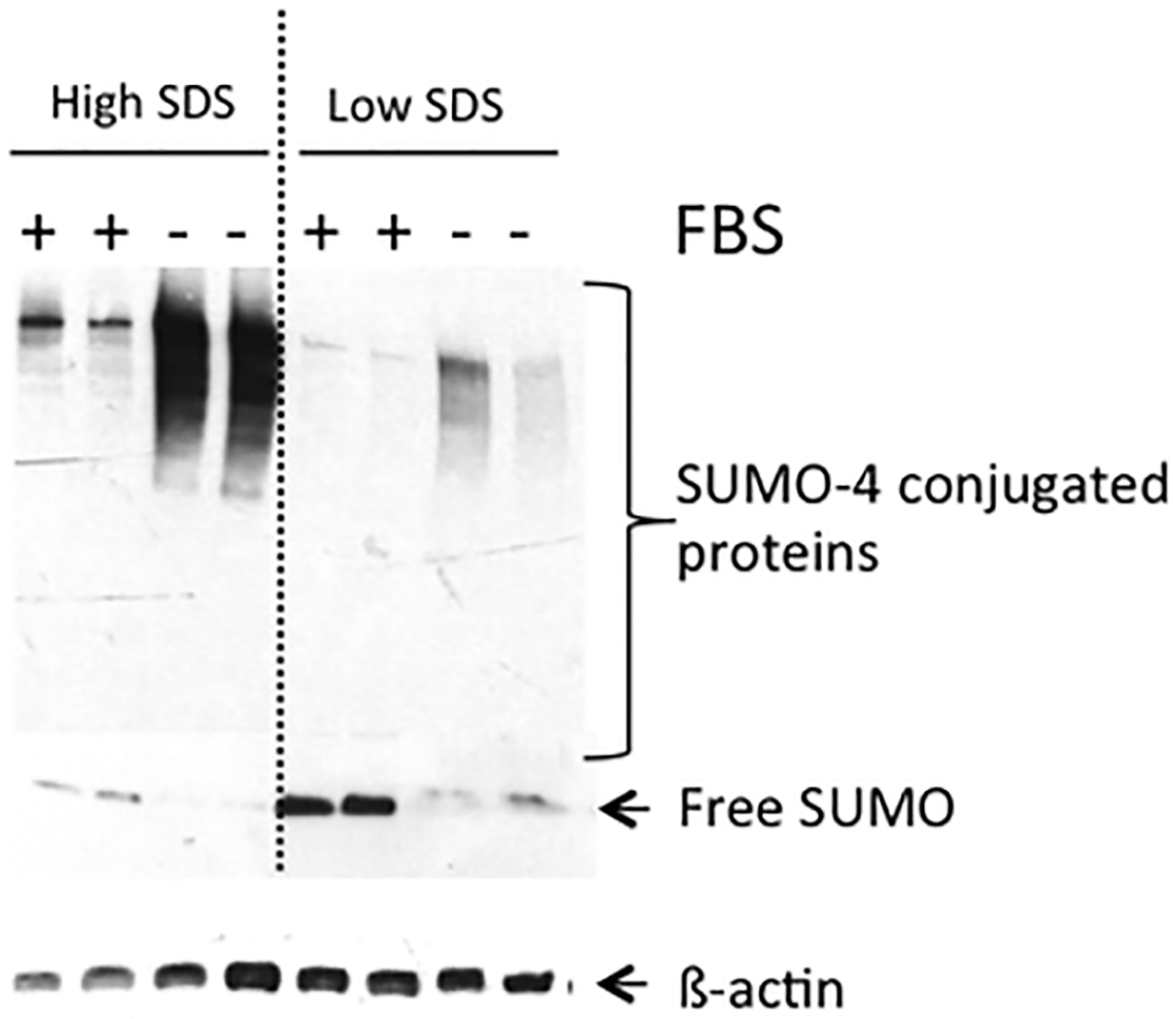
SUMO-4 covalently interacts with its targets in BeWO cells. BeWo cells were stressed by fetal bovine serum (FBS) depravation (24h) to induce global SUMOylation. Elevated levels of SUMO-4 conjugated proteins were observed in high SDS buffer, but not in the low SDS buffer preparations, suggesting that SUMO-4 protein interactions are covalent in nature.

After inducing global SUMOylation in BeWo cells by FBS deprivation for 24h, SUMO-4 levels increased by 2.6±0.5 fold (n = 3, p<0.05; data not shown). Elevated levels of SUMO-4 conjugated proteins were observed in high SDS buffer, but not in the low SDS buffer preparations suggesting that SUMO-4 protein interactions are predominantly covalent in nature (Fig 6).

## Discussion

We report the novel finding of the SUMO-4 isoform in the human placenta. SUMO-4 was present in the villous trophoblast layer throughout gestation. While SUMO-4 mRNA and protein expression remains unchanged across gestation, hyper-SUMOylation occurred in PE placentas. Oxidative stress, simulated by H_2_O_2_ treatment of BeWo cells, Jar cells and first trimester placental explants was able to induce *SUMO-4* mRNA, as well as SUMO-4 protein translation. Similarly, hypoxia-reperfusion injury of first trimester placental explants also increased SUMO-4 expression and protein conjugation. By comparison *SUMO-1* to *SUMO-3*, *SENP2* and *UBC9* upregulation was less pronounced and less consistent amongst models. Lastly, SUMO-4 was found to bind to its targets in a covalent manner.

The severe early PE placenta is characterized by placental villous trophoblast stress, largely due to hypoxia-reperfusion injury induced oxidative stress that suppresses translation of key proteins, such as placental growth factor (PlGF) [18]. The underlying pathogenesis is due to diseased spiral arteries [19] and may be accompanied by infiltration of maternal leukocytes [20]. Hypoxia has been shown to enhance placental SUMOylation *in-vitro* [11], which emphasizes the importance of SUMOs in the stress response pathway underlying PE. SUMOylation has been shown to specifically alter key transcription factors involved in PE placental dysfunction, including glial cell missing-1 (GCM-1) [16,21], downstream regulatory element antagonist modulator (DREAM) [22,23], hypoxia inducible factor-1α (HIF-1α) [24,25] and the downstream product PlGF [18], that is now used as a diagnostic test for pre-eclampsia [26]. GCM-1 is a transcription factor involved in the terminal differentiation of villous cytotrophoblasts into the outer syncytiotrophoblast later that secretes PlGF. Hyper-SUMOylation of GCM-1 was reported in PE, leading to repressed GCM-1 levels, thus, contributing to reduced trophoblast differentiation [16,27] and thus reduced PLGF output [18]. Conversely, SUMOylation increases the stability and activity of the transcription factor DREAM [22,24]. In the placenta, DREAM acts to decrease the expression of GCM-1 and is upregulated in PE [23]. Lastly, in first trimester tissue, HIF-1α is associated with SUMO-2/3, which alters HIF-1α stability. In early PE, SENP has been shown to cause de-SUMOylation of HIF-1α, thereby increasing its activity [25]. Collectively, these studies demonstrate that SUMOylation is altered in PE, however further confirmatory studies are required to determine the exact role of the SUMO-4 isoform within the disease.

Early investigators of SUMO-4 doubted its functional role in various cell types. However, it is now known that SUMO-4 is involved not only in cell cycle progression and apoptosis, but also in intracellular stress response signaling [28]. SUMO-4 was also previously thought only to be present in the kidneys, pancreas and immune cells [6–8]. However, this study has identified SUMO-4 expression in the human placenta, using several models including cell lines and placental tissue. SUMOylation can occur by covalent and non-covalent interactions. Covalent interactions of a SUMO isoform with its target may disrupt or prevent other potential downstream interactions from occurring. By contrast, when SUMO associates non-covalently with a target protein SIM/SBM binding motif, this interaction may allow for the additional interaction of a subsequent protein [4]. Further research to determine which target proteins SUMO-4 interacts with in a covalent manner is now required.

One of the limitations of this study includes the difficultly of estimating the extent of placental tissue oxidative stress and ischemic-reperfusion injury found in PE *in-vivo*. Cellular responses to H_2_O_2_ have also shown to vary significantly depending on concentration, mode of production (endogenous or exogenously administered) and cell type exposed [29]. At this point, our data is insufficient to determine if the SUMO-4 hyperSUMOylation is driven by increased conjugation, via UBC9, or deconjugation, via SENP2. The mRNA levels of these two enzymes varied in our treated cell and explant models. We did not measure the activity of UBC9 or SENP2, which have previously been shown to be differentially affected by reactive oxygen species in a dose-dependent manner [29,30]. It is also important to note that SENP, in addition to deconjugation activity, is involved in processing of the SUMO precursors [31]. However, despite these limitations, our data demonstrated that not only did SUMOylation of SUMO-4 increase oxidative and hypoxic stress in first trimester explants, but also these effects are congruent with increases that occurred in PE placentas. At this time, it is unknown whether these effects would be sustained in the long-term or are simply an acute cellular stress response.

Functional redundancies do exist between SUMO isoforms. Some insight into this concept has been gained through the use of transgenic mouse models. SUMO-1 knockout mice do not show overt changes during embryonic development and have a relatively maintained homeostatic balance. However, compensatory SUMOylation by SUMO-2/3 does occur in SUMO-1 null mice [32]. Similarly, SUMO-3 null mice are also phenotypically normal [33]. Strikingly, homozygous SUMO-2 knockout mice showed embryonic lethality as early as day 10.5. These knockouts had impaired chorion allantoic fusion, a key step in mid-gestation placental development in mice. In support for a key regulatory role of SUMO-2 in placental development, the heterozygous knockout mice for SUMO-2 demonstrate severe intrauterine growth restriction [33]. Currently no SUMO-4 knockout models have been developed to test its functional significance *in-vivo*. Whether or not SUMO-4 is dispensable due to compensatory effect of other SUMO family members is not presently known.

The coordinated addition and removal of SUMOs to target proteins is a fundamental post-translational mechanism involved in a variety of cellular processes that may be disrupted in specific pathological conditions. We have now demonstrated that SUMO-4 is stably expressed in the human placenta throughout gestation. Our data suggest that hyper-SUMOylation, especially that of SUMO-4 may be involved in the pathogenesis of PE, a major obstetrical complication affecting up to 8% of all pregnancies [34]. To date, the only known intervention commonly accepted for the prevention of PE is low-dose aspirin [35]. However, identification of the SUMO-4 isoform in the trophoblast of the placenta, which is altered in preeclamptic placentas, may provide a novel target for therapeutic intervention. Since, the most severe cases of PE result in preterm birth with intrauterine growth restriction, new efforts to create drugs that target the underlying etiology of trophoblast placental dysfunction in PE are needed. As a result, addressing the SUMO pathway may be one such mechanism to focus on.

## Supporting information

S1 Fig Primary data for Fig 1 - SUMO-4 mRNA and protein levels across gestation and in pre-eclampsia (PE). SUMO-4 (A) mRNA (n = 8–15) and (C) protein (n = 3–6) expression was unchanged across gestation. Conversely, PE placentas showed elevated SUMO-4 (B) mRNA (**p<0.01, n = 4–6) and (D) protein (*p<0.05, n = 11–13) compared to pre-term age matched controls (PTC). Raw expression values. 1st = first trimester, 2nd = second trimester. Values represented as mean+SEM.
(TIF)

S2 Fig Primary data for Fig 3A and 3C. Please see respective figure legends for full figure descriptions.
(TIF)

S3 Fig Primary data for Figs 4A and 5A. Please see respective figure legends for full figure descriptions.
(TIF)

## Abbreviations

LMWH: low molecular weight heparin
PE: pre-eclampsia
PLGF: placental growth factor
SENPs: sentrin-specific proteases
SUMO: small ubiquitin-like modifiers
