# Supplementary figures and images for: SUMO-4: A novel functional candidate in the human placental protein SUMOylation machinery
Source: PLoS One. 2017 May 17;12(5):e0178056. doi: 10.1371/journal.pone.0178056 (PMC5435238; doi:10.1371/journal.pone.0178056)

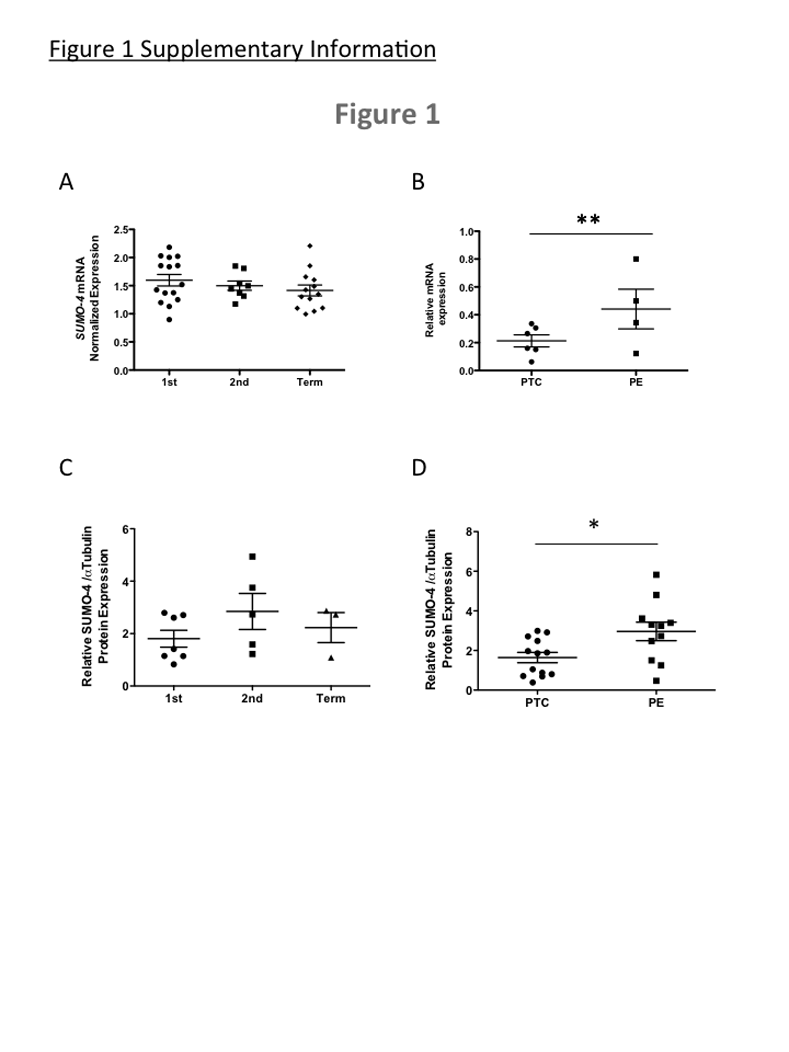

Supplement: S1 Fig — SUMO-4 (A) mRNA (n = 8–15) and (C) protein (n = 3–6) expression was unchanged across gestation. Conversely, PE placentas showed elevated SUMO-4 (B) mRNA (**p<0.01, n = 4–6) and (D) protein (*p<0.05, n = 11–13) compared to pre-term age matched controls (PTC). Raw expression values. 1st = first trimester, 2nd = second trimester. Values represented as mean+SEM. (TIF) [file pone.0178056.s001.tif]

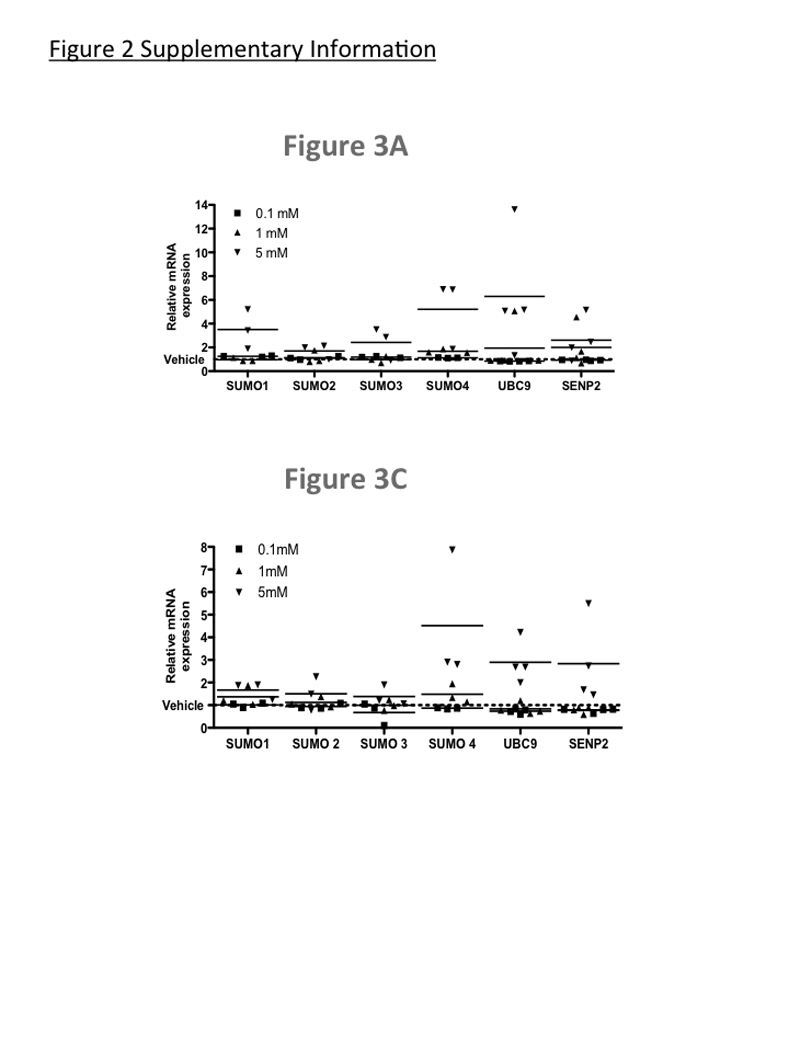

Supplement: S2 Fig — Please see respective figure legends for full figure descriptions. (TIF) [file pone.0178056.s002.tif]

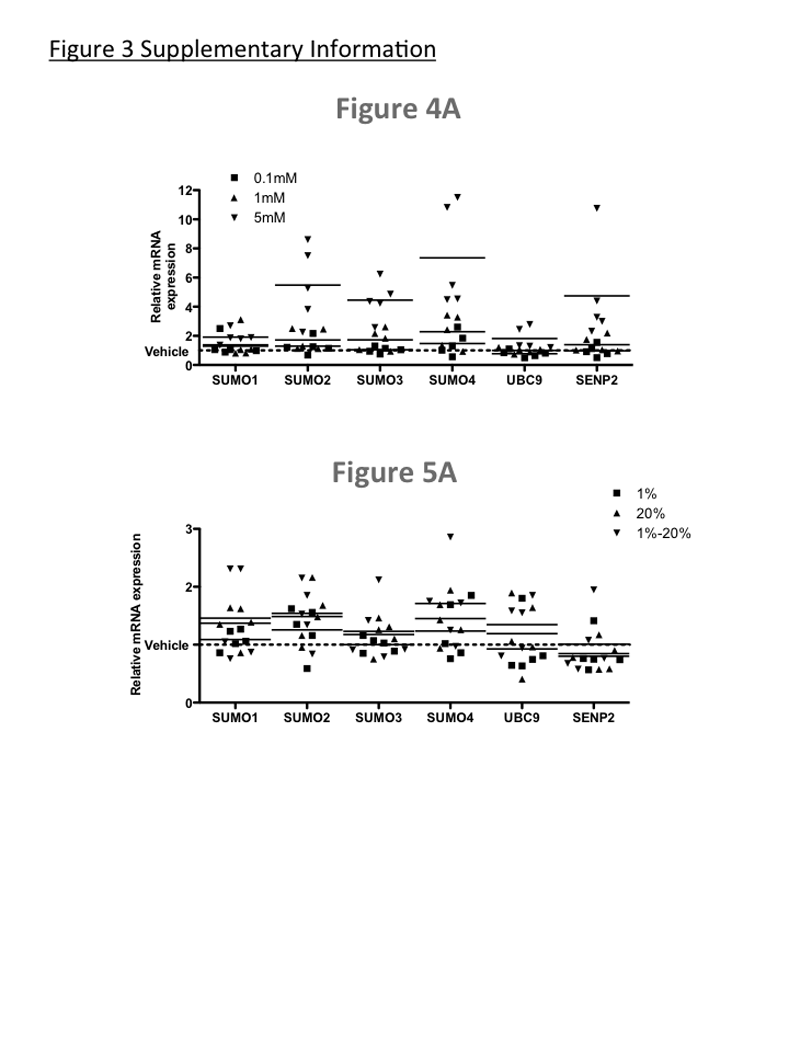

Supplement: S3 Fig — Please see respective figure legends for full figure descriptions. (TIF) [file pone.0178056.s003.tif]
